# Effects of screw configuration and interfacial properties on oil incorporation in high moisture extrusion

**DOI:** 10.1016/j.crfs.2025.100989

**Published:** 2025-01-31

**Authors:** Shuzo Hashimoto, Naoya Ikenaga, Atze Jan van der Goot, Leonard M.C. Sagis

**Affiliations:** a Laboratory of Physics and Physical Chemistry of Foods, Wageningen University & Research, Bornse Weilanden 9, 6708 WG, Wageningen, the Netherlands; b Laboratory of Food Process Engineering, Wageningen University & Research, Bornse Weilanden 9, 6708 WG, Wageningen, the Netherlands; c Fuji Oil Global Innovation Center Europe, Bronland 10, 6708 WH, Wageningen, the Netherlands; d Fuji Oil Co., Ltd., 1 Sumiyoshi-cho, Izumisano-shi, Osaka, 598-8540, Japan

**Keywords:** High moisture extrusion, Oil incorporation, Screw configuration, Kneading discs, Large amplitude oscillatory shear, Multiphoton microscopy

## Abstract

Routes to include oil in meat alternative products made through high moisture extrusion were investigated. We investigated effects of screw configuration and oil-water interfacial properties of the incorporated emulsion on the behavior of the oil during extrusion, and the characteristics of the extrudate after extrusion. Oil was incorporated in the form of an emulsion, and for comparison also directly added without prior emulsification. For oil addition by emulsion, several plant-based protein sources were compared as emulsifier. The choice of protein emulsifier had a strong effect on wedge length of the extrudate, the maximum linear strain and value of G′ in shear, and the rupture strength of the extrudate. Two screw configurations were used. One with 1 kneading disc section and another with 4 kneading disc sections. It was found that fewer kneading discs in the screw led to less mechanical energy input, leading to less shear on the dough in the extruder. The use of fewer kneading elements resulted in less oil leakage from the product, while the product also was stronger, as demonstrated by the higher rupture strength in the texture analysis, and higher storage modulus G’ in oscillatory shear rheology. Less deformation of the air pockets between protein structures could be seen in the screw with fewer kneading discs in multiphoton microscopy. As a result, both the screw configuration and the interfacial properties of the plant protein emulsifier need to be considered when developing meat alternative products containing oil as a route to control the texture of these products.

## Highlights

• Plant-based emulsions were incorporated in high moisture extrudates.
• Type of protein emulsifier had a strong effect on wedge length of extrudates.
• Maximum linear strain and G′ in the LVE-regime were also affected.
• More kneading discs resulted in more oil leakage from the product.
• More kneading disks resulted in lower rupture strength and lower G′.

## Graphical abstract

**Figure:**
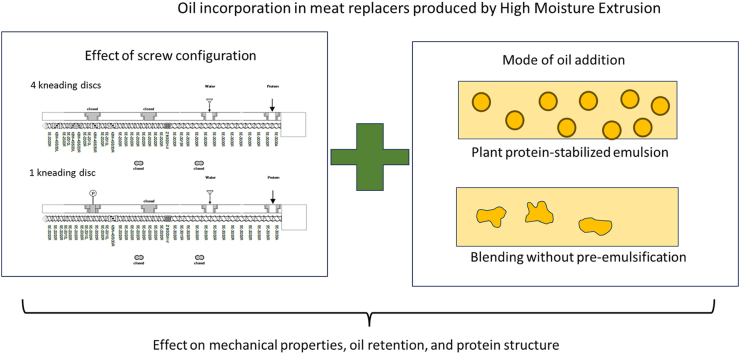

## 1 Introduction

The world's population is expected to grow up to 10 billion people by 2050 according to FAO (Hayes, 2023). A substantial proportion of many people's protein intake originates from animals (Popkin et al., 2012; Aiking, 2011), and a (partial) transition from consumption of animal-based towards plant-based products is one of the solutions to lower the environmental impact of current diets (De Bakker and Dagevos, 2012). This explains why plant protein is gaining interest as an alternative source of protein, especially textured vegetable protein products (Joseph et al., 2020). The most widely applied technology to create a meat-like fibrous structure is high moisture extrusion (HME) (Akdogan, 1999; Cheftel et al., 1992; Schmid et al., 2022; Dekkers et al., 2018). A number of plant proteins are well-suited for the HME process, for example, soy protein (Wittek et al., 2021a), pea protein (Osen et al., 2014), wheat gluten (Pietsch et al., 2017), faba protein (Saldanha Do Carmo et al., 2021), mung bean protein (De Angelis et al., 2023), although in practice mainly proteins from soy, pea and wheat are used.

Since fat in real meat contributes significantly to the sensory properties of meat, the incorporation of oils and fats in meat alternatives is probably essential. The intramuscular fat structure has an important role in food sensory and quality properties such as appearance, texture, juiciness, and mouthfeel (Sun et al., 2018; Bourouis et al., 2023; Choi et al., 2019). However, it is not trivial to incorporate much oil into products made using high moisture extrusion, because oil leaking and wall slip may occur at high levels of oil addition (6–8% (Ikenaga et al., 2025)). But previous research also suggested that emulsified oil can improve the structure of products compared with direct oil addition (Non-emulsified addition)(Wang et al., 2022). The main challenge is to prevent oil from being expelled from the product, which could be done by adding oil via small droplets, as shown previously (Ikenaga et al., 2025). However, extreme shear forces in the extruder can break droplets and cause coalescence, leading to oil being expelled from the product (Ikenaga and Sagis, 2024).

We here hypothesize that the screw configuration together with the mechanical properties of the interface between water and oil, plays an important role in whether oil is kept in the product or will be expelled. The number of kneading discs in the screw is an important factor, since these discs are thought to have an important role in food extrusion, especially in protein texturization (Wu et al., 2023). The role of kneading discs is to properly disperse the ingredients, but they also generate heat via viscous dissipation (Andersen; Köllmann et al., 2024). Therefore, the number of kneading discs has a large influence on the properties of extrudates. The kneading discs may also increase the shear forces on oil droplets. Depending on the mechanical properties of the oil-water interface of the droplets, the shear forces may lead to destabilization of the interfacial film, and hence to coalescence of droplets, followed by expelling of oil from the structure. The mechanical properties of the interface can be controlled by an appropriate choice of emulsifier. However, there is no research focusing on the synergy between screw configuration and the surface properties of the oil droplets (particularly the surface rheological properties in the large deformation or nonlinear viscoelastic (NLVE) regime).

In this paper, we will discuss the results of experiments that combine the kneading disc effect and interfacial properties on retention of oil in the extrudate, and on the mechanical properties and structure of the extrudate. Specifically, we have compared the mechanical properties (in both shear and texture analysis), oil retention, and protein structure of soy-based extrudates containing oil, produced with HME, for two different screw configurations (1 versus 4 kneading disks). We also evaluated the extrudate properties for three plant-based emulsifiers and compared addition of oil in a pre-emulsified state, with simply blending the oil with the soy protein in the feed.

## 2 Materials and methods

### 2.1 Materials

Medium-chain triglyceride (MCT) oil (MIGLYOL® 812N) was purchased from IOI Oleochemical (France). Commercial potato protein isolates (POPI), POPI-1, and POPI-2 were kindly donated by Avebe U.A. (Netherlands). A commercial pea protein isolate (PPI), was purchased from Roquette Frères (France). A commercial soy protein concentrate (SPC), ALPHA® 8, was purchased from Solae Europe S.A. (Switzerland). Details of the protein isolates on protein content, average molecular weight, and degree of processing of the isolates are given in Table 1. The interfacial rheological properties of the Potato protein and Pea Protein were quantified following Ikenaga and Sagis (2024) (Ikenaga and Sagis, 2024) and are summarized in Table 2. Milli-Q water (PURELAB® Ultra Water Purification System, Germany) was used for emulsion preparation and analyses. Hydrochloric acid (HCl), sodium hydroxide (NaOH), sodium chloride (NaCl), 99.5% ethanol, and Rhodamine B were purchased from Merck (Germany). Petroleum ether 40–60 °C was purchased from Actu-All Chemicals B.V. (Netherlands). BODIPY 505/515 was purchased from Thermo Fisher Scientific (Netherlands).

**Table 1:** Details of the protein isolates. Protein content was measured by the Dumas method, and weight average molecular weight was shared by the suppliers. The protein content of all protein powders was determined by the Dumas method (conversion factor: 5.5).

Supplier	Source	Protein name	pH	Protein content (wt%)	Weight average molecular weight (kDa)
Avebe	Potato	POPI-1	Neutral	77.18	80
		POPI-2	Acidic	77.67	32
Roquette	Pea	PPI	Neutral	69.36	94

**Table 2:** Summary of interfacial rheological properties of plant proteins by Ikenaga and Sagis (2024). Here Ed’ is the dilatational storage modulus, and G_s_' is the interfacial shear storage modulus. LAOD means large amplitude Oscillatory Dilatation, and LAOS means large amplitude oscillatory shear. *G*_*s*_*’* listed in the LAOS column is the average of 5 points in the LVE regime.

	LAOD		LAOS
Protein name	*Ed*’ (mN/m)	Qualitative description of behavior	*G*_*s*_*'* (mN/m)
POPI-1	20.0	Soft/Stretchable	200.0
POPI-2	29.2	Stiff/More brittle	121.4
PPI	13.4	Soft/Stretchable	32.3

### 2.2 Methods

#### 2.2.1 Emulsion preparation

Protein ingredients (POPI-1, POPI-2, and PPI) were dissolved in Milli-Q water at a protein concentration of 1.8–1.9 wt%, based on Dumas results. The solutions were stirred at 20 °C for 4 h, then at 4 °C for 20 h. The pH and conductivity of the solutions were adjusted using 1.0 M HCl, 1.0 M NaOH, and NaCl to pH 3.6 (only for POPI-2)/pH 7.0 (for the others) and 1500 μS/cm. After that, the protein content was adjusted to 1.76 wt% by adding Milli-Q water. Protein solutions were stored at 4 °C until they were used.

Emulsions with 15% MCT oil content were prepared with the protein solutions. The mixtures were first pre-homogenized using an L5M-A Silverson laboratory mixer with a 1 mm screen hole (Silverson Machines Ltd., United Kingdom) at 5000 rpm for 5 min. Then, the mixture was homogenized by a GEA Lab Homogenizer PandaPLUS 2000 (GEA Group AG, Germany). The beakers for the mixtures before and after homogenization were kept in ice water during homogenization to prevent excessive heating. The pressure and the number of passes for the homogenization are shown in Table 3. The emulsions were finally collected in a blue cap bottle and stored overnight at 4 °C before analysis. The particle size distribution was measured and confirmed that it had a main peak at 0.9–1.0 μm (data not shown).

**Table 3:** Conditions for homogenization (adjusted to get similar droplets sizes).

Emulsifier	Oil content of emulsion (wt%)	Pressure (bar)	The number of passes
POPI-1	15	150	6
POPI-2	15	110	6
PPI	15	110	8

#### 2.2.2 Extrusion experiments

**Fig. 1:**
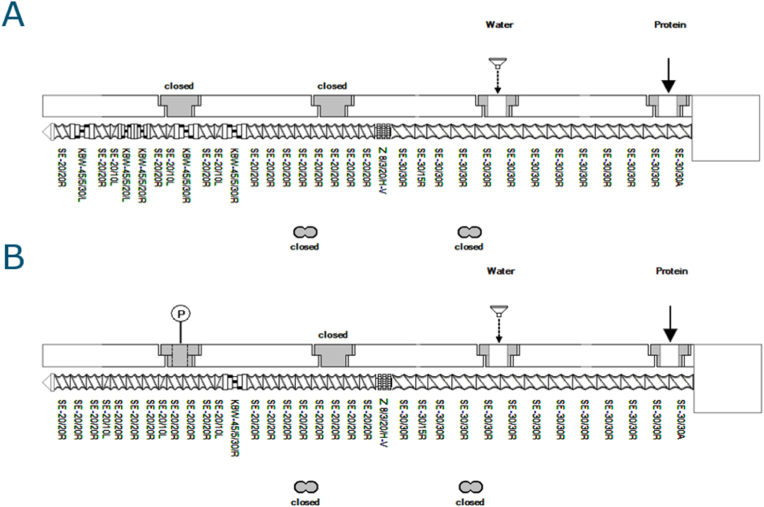
Two screw configurations were tested. Screw A (4KD) includes 4 kneading discs and is the same screw as described by Nieuwland et al.(2023) (Nieuwland et al., 2023). Screw B (1KD) includes 1 kneading disc. The incorporated screw elements are SE: forwarding element Z: mixing element KBW: kneading disc.

The extrusion experiments were performed using a co-rotating twin-screw extruder, TwinLab-F 20/40 (Brabender GmbH, Germany), with an L/D ratio of 40 and a screw diameter of 20 mm, as described by Nieuwland, Heijnis, van der Goot, and Hamoen (2023) (Nieuwland et al., 2023). Three 100 mm blocks with a 25 × 7 mm geometry were attached as a cooling die after the main barrel of the extruder. We used 2 screw configurations. The 1st configuration includes 4 kneading-disc zones, making it the same screw as described by Nieuwland et al.(2023) (Nieuwland et al., 2023) (Fig. 1A). The 2nd configuration includes 1 kneading-disc zone only (Fig. 1B). In this paper, we refer to the 1st screw as 4KD (KD: Kneading Disc) and the 2nd screw as 1KD. A pressure sensor and temperature sensor were attached to the die plate. SPC was dosed via a Brabender Screw Feeder DDSR20 (Brabender GmbH, Germany) at the inlet located at 0D. Here, D is the diameter of the screw, and the number in XD describes the distance from the inlet in multiples of D. For the non-emulsion test, we prepared a pre-mixed powder with SPC and MCT oil, after which the pre-mixed powder was added via the Brabender Screw Feeder DDSR20. A pre-mixed powder was prepared by Varimixer planetary mixer-RN20 VL-2 (Varimixer A/S, DK). The same extrusion settings of temperature and screw speed as described by Nieuwland et al. (2023) (Nieuwland et al., 2023) were applied for all experiments in this study.

The extrudates were cut on a roller using scissors. All formulations for the extrudate with/without emulsions are shown in Table 4. The extrusion experiments were performed twice. Pressure, temperature at the cooling die, and specific Mechanical energy (SME) were measured. The average of pressure, temperature at the cooling die, and SME within one trial were calculated. After that, each data was combined (n = 2).

**Table 4:** Formulations of the extrudate with/without emulsions. To fix the solid content for SPC (Soy protein Concentrate), the percentage of SPC was slightly adjusted between samples. POPI-1 refers to the emulsifier Potato Protein Isolate-1. POPI-2 refers to Potato Protein Isolate-2. PPI means Pea Protein Isolate. Emulsion composition and preparation are described in Table 3.

Extrudate Sample name	SPC	POPI-1	POPI-2	PPI	Non-emulsion
SPC (%)	43.3	43.3	43.3	43.3	43.3
Water (%)	56.7	–	–	–	48.25
Emulsion (%)	–	56.4	56.4	56.4	–
MCT (Not emulsified) (%)	–	–	–	–	8.45
pH of emulsion	–	7.0	3.6	7.0	–
Conductivity of emulsion (μS/cm)	–	1500	1500	1500	–
Oil in extrudate from emulsion (%)	–	8.45	8.45	8.45	–
Oil in extrudate from MCT (%)	–	–	–	–	8.45

#### 2.2.3 Dry matter content

The extrudate pieces were collected just after they came out of the cooling die. The weight of the extrudate pieces was immediately measured. Extrudate pieces were put into an oven and were subject to air drying at a temperature of 95 °C overnight (∼15 h). The solid content of all extrudes was determined by averaging the measurements of four extrudate pieces each, from two replicated extrusion experiments (n = 8 in total). Dry matter data is shown in Fig. S3.

#### 2.2.4 Oil content and die leakage

The oil drops leaking from the outlet of the cooling die during the extrusion experiments were collected in a plastic cup for approximately 10 min. The cups with leaked oil were dried in an oven at 40 °C overnight and the remaining oil in the cup was measured (n = 4). The rate of oil leakage $R_{\text{ol}}$ (g/h) was calculated using:

(1) $$R_{\text{ol}}=\frac{W_{\text{oil}}\times 3600\;}{t_{\text{oc}}\;}$$

Here $W_{\text{oil}}$ is the weight of oil (g), and $t_{\text{oc}}$ is the collection time (s).

For the oil content of the extrudate, 10 g of extrudate was cut and collected in an aluminium cup. After measuring the weight, the cut extrudate pieces were freeze-dried. Then, the dried extrudate pieces were ground using a mortar and muller into a powder. The oil was extracted using a fully automated Soxhlet system, Southern, (C. Gerhardt GmbH & Co. KG, Germany) with petroleum ether 40–60. The ether was evaporated, and the extracted oil was weighed (n = 4). The oil content was calculated with Eq. (2). Data is shown in the supplemental figures (Fig. S1).

(2) $$Oil\;content\;\left(\%\right)=\frac{Extracted\;oil\;\left(g\right)\times 100}{Weight\;of\;extrudate\;pieces\;\left(g\right)}$$

#### 2.2.5 Rupture strength by blade cutting

The rupture strength was measured at room temperature in two directions, Ft and Fl (Fig. 3), using a texture analyzer TA.TX plus with a blade with 60° angle at the edge(Warner Bratzler) (Stable Micro Systems Ltd, United Kingdom). The extrudate was cut into a piece with a size of 20 mm long and 25 mm width (Fig. 5A). The maximum force during the strain 0–80% with a test speed of 1 mm/s was recorded in both directions, as Ft and Fl for each, using a trigger force of 0.5 N (n = 12).

#### 2.2.6 Wedge length

The wedge length was determined through measuring the length between the apex of the wedge to the end of the wedge. The extrudates were split in the middle, horizontally on the long side, in the sample flow direction, and pictures of the wedge were taken. The wedge length was then taken as the average of the wedge length for the four wedge shapes each from two replicated extrusion experiments (n = 8 in total).

#### 2.2.7 Multiphoton excitation microscopy

The samples were cut in the middle following the direction of flow and their cross-sections were dyed (Fig. S2A). The dye solution was prepared just before use by mixing 0.25 mg/mL BODIPY 505/515 solution and 0.04 mg/mL Rhodamine B solution in a 1:1 ratio. The cut samples were dyed in a dark place at 5 °C for 15 min. Then, the dyed surface was washed twice with Milli-Q water carefully and observed using a multiphoton excitation microscopy Leica SP8Dive (Leica, Germany) with a HC PL IRAPO 40x/1.10 Water objective. The laser excitation wavelength was set at 1000 nm (the power at 1.2%), and the emission range for BODIPY 505/515 and Rhodamine B were 500–550 nm and 590–650 nm (the gain at 20% for both). Images (900 μm × 1500 μm) of the cutting surface were taken (Fig. S2B). The image was extracted at 6–8th and 12–14th field of view (FOV) (Fig. S2B), which were counted from the top surface of the cross-section that was divided into 30 FOV of the microscope. For both layers, 4 merged images of the area where fluorescence was visible were obtained by the merge images function (Multiphoton mode).

Merged images were exported in greyscale using the software LAS X (Leica, Germany) for analysis using the image processing software ImageJ. Then air pockets between protein structures were extracted. For the image analysis process, we removed the unnaturally large air pockets which clearly consisted of multiple partially merged air pockets and focussed only on isolated pores. We extracted air pockets in the range of 50–10000 μm^2^ to obtain the diameter of their major and minor axis, their orientation angle, their perimeter, and their area. The deformation value *D* was calculated using the diameter of the major axis, *a*, and the diameter of the minor axis, *b*, with Eq. (3) (n = 8).

(3) $$D=\frac{a-b}{a+b}$$

The value of D is 0 for a perfectly spherical pore, and equal to 1 for a completely stretched one.

#### 2.2.8 Rheological properties

The rheological properties were measured with a closed cavity rheometer (CCR) (RPA elite, TA instruments, New Castle, Delaware, USA). We measured the rheological properties of both the blend of ingredients of the extrudate and the extrudate itself. For both processes, the closing pressure was 4.5 bar to prevent water evaporation during heating. The closed cavity rheometer has a radius of 22.5 mm. The maximum height of the inner cavity is 4 mm in the bi-conical cylinder where the surface has radial serrations for the prevention of slipping. The angle of the cylinder is 3.35° (Schreuders et al., 2020, 2022).

Blends of SPC and oil were made by preparing the emulsions as described in section 2.2.2 and mixing it with SPC. The ratio between SPC and the emulsion was the same as the ratio in the extrusion trial, which is described in Table 4. After mixing, the powder was kept in a closed plastic container for 20 min. Then, approximately 6 g of mixed ingredients (dough) was placed into the cavity (Schreuders et al., 2021). During the rheological measurement, the dough was rested first by keeping it at 35 °C for 2 min without shear. Then, we performed a heating process that imitates the extrusion temperature conditions. Heating was done by increasing the temperature from 35 °C to 120 °C in 15 min. After this process, the sample was cooled from 120 °C to 35 °C. For both heating and cooling processes, we set the amplitude and frequency in the lower range to minimize the effect of shear. The amplitude was 0.14%, and the frequency was 0.1 Hz. After the heating and cooling step, the sample was left in the closed cavity for 15 min. Next, we performed a strain sweep test (0.01–1000%, at 35 °C, frequency at 1 Hz) (n = 3).

To measure the rheological properties of the extrudates, samples were made by cutting extrudates into 25 mm × 25 mm X 7 mm pieces. These dimensions were selected because the product exiting the die measures 25 mm × 7 mm. Samples were covered with plastic film and inserted into the cavity of the CCR. Prior to the rheological measurement, the extrudate was stabilized by keeping it at 35 °C for 2 min. After the stabilizing process, a strain sweep test (0.01–1000%, at 35 °C, frequency at 1 Hz) was performed (n = 6).

The storage modulus(G’) in the linear viscoelastic regime was taken as a measure for stiffness. The maximum linear strain was defined as the strain where the decrease of G′ from its constant value in the LVE reached 5% (Xia et al., 2021).

#### 2.2.9 Large amplitude oscillatory shear(Laos) analysis

The torque (Nm) and strain (%) data obtained from the LAOS measurements in CCR were used for the analysis of the response in the NLVE regime. MITlaos software (Version 2.1 beta, freeware distributed from MITlaos@mit.edu) was used with a similar protocol as reported by Schreuders et al. (2021). We also made Lissajous curves using the MITlaos software, and used these to compare samples based on the occurrence of strain softening/hardening behavior, and their transition to plastic behavior (Ewoldt et al., 2008; Ewoldt and McKinley, 2010).

#### 2.2.10 Statistical analysis

In this study, IBM SPSS Statistics 22 (IBM SPSS Inc., USA) was used to perform One-way ANOVA with Tukey HSD when we evaluated the significant difference between 3 extrudates with emulsion and extrudates with non-emulsified MCT oil. The significance was defined as *P* < 0.05 and significant differences were marked with different letters.

When we evaluated the significance between 4KD and 1KD screw, we used the t.test function in Microsoft Excel. The significance was defined as *P* < 0.05 and *P* < 0.01 and significant differences were marked with different number of stars.

## 3 Results and discussion

### 3.1 Extrudate analysis

Extrudates as described in Table 4 were produced using the two screw configurations (Fig. 1). The extrudates containing oil in the form of non-emulsion had almost the same solid content as the extrudates containing oil in the form of emulsion (Fig. S3). All extrudates were further analyzed with respect to oil leakage, wedge length, rupture strength, and shear rheological properties.

#### 3.1.1 Oil leakage at the cooling die

Oil leakage from the product when it exited the die was observed in several extrudates. Given the fact that the moisture content is the same (Fig. S3), the difference in amount of oil leakage is most likely due the difference in the emulsifier properties. For the samples in which oil was added as an emulsion, produced with the 4KD screw configuration, there is no significant difference between extrudates prepared with POPI-1 or POPI-2 stabilized emulsion. The use of PPI led to the lowest amount of oil loss, and was significantly lower than in case POPI-2 was used. This is in spite of the fact that POPI-2 produces oil-water interfaces with a much higher dilatational and interfacial shear modulus than PPI, and are hence much stiffer (Table 2). (Ikenaga and Sagis, 2024) There is no significant difference between adding the oil in non-emulsion form or adding the oil as a POPI-1 or POPI-2 stabilized emulsion, for the 4KD screw.

**Fig. 2:**
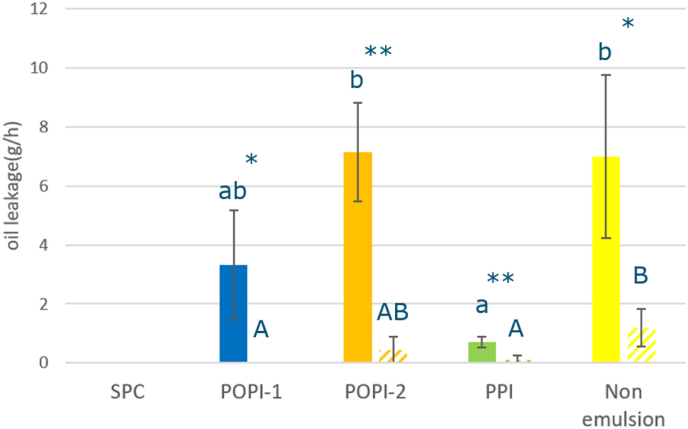
Oil Leakage at the cooling die (solid bar: 4KD, shaded bar: 1KD; SPC: gray, POPI-1: blue, POPI-2: orange, PPI: green, Non-emulsion: yellow); Statistical analysis: One-way ANOVA, Tukey HSD test, P<0.05, for comparing samples within one screw configuration; samples which are significantly different are marked with different letters (1KD: A,B,C and 4KD: a,b,c); t.test for comparing differences between screw configurations: ∗: P<0.05, ∗∗: P<0.01).

Fig. 2 shows that oil leakage in the 1KD screw is significantly lower than oil leakage in 4KD for all the extrudates. As we noted before, adding additional kneading discs in the screw configuration increases the shear forces on the droplets, which led to more coalescence and expelling of oil. Within the 1KD samples, POPI-1 and PPI showed significantly less oil leakage than the Non-emulsion, with POPI-1 even showing no oil leakage.

**Fig. 3:**
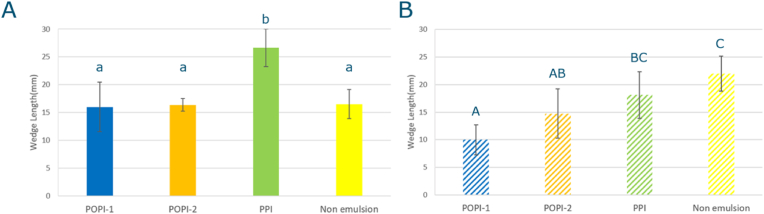
Wedge length of extrudates, A: wedge length of 4KD extrudate, B: wedge length of 1KD extrudates, with solid bar: 4KD, shaded bar: 1KD, POPI-1: blue, POPI-2: orange, PPI: green, and Non-emulsion: yellow. Statistical analysis: One-way ANOVA, Tukey HSD test, P<0.05; samples which are significantly different are marked with different letters (1KD: A,B,C and 4KD: a,b,c).

#### 3.1.2 The effect of screw configuration and oil addition on wedge length

**Fig. 4:**
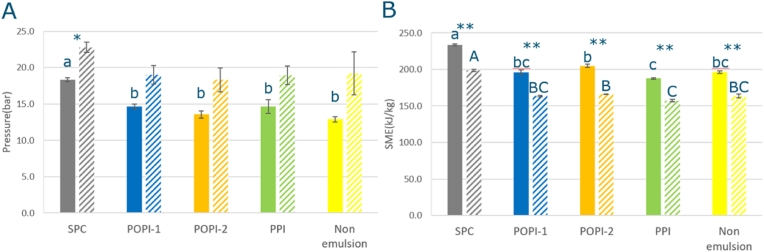
Drive condition parameters for both screw configurations. A: pressure (bar) at the die plate, B: SME (Specific Mechanical Energy), solid bar: 4KD, shaded bar: 1KD, POPI-1: blue, POPI-2: orange, PPI: green, and Non-emulsion: yellow. Statistical analysis: One-way ANOVA, Tukey HSD test, P<0.05, for comparing samples within one screw configuration; samples which are significantly different are marked with different letters (1KD: A,B,C and 4KD: a,b,c); t.test for comparing differences between screw configurations: ∗: P<0.05, ∗∗: P<0.01).

The wedge length for both 1KD and 4KD are shown in Fig. 3. The results imply that oil leakage (Fig. 2) and drive conditions of the extrusion (Fig. 4) both affect the wedge length.

**Fig. 5:**
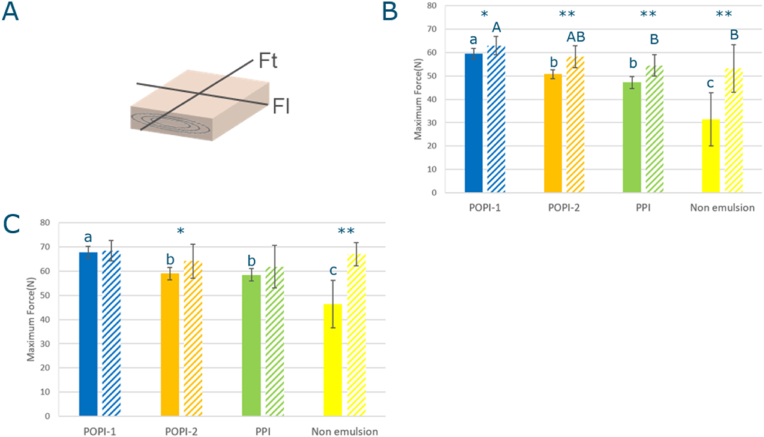
Texture analysis of the extrudates. A: cutting directions in texture analyzer B: Ft (maximum rupture strength in the fiber direction) C: Fl (the maximum rupture strength in the direction perpendicular to the fibers), Solid bar: 4KD, shaded bar: 1KD, POPI-1: blue, POPI-2: orange, PPI: green, and Non-emulsion: yellow. Statistical analysis: One-way ANOVA, Tukey HSD test, P<0.05, for comparing samples within one screw configuration; samples which are significantly different are marked with different letters (1KD: A,B,C and 4KD: a,b,c); t.test for comparing differences between screw configurations: ∗: P<0.05, ∗∗: P<0.01).

Fig. 4 shows the effect of different screws on the SME and pressure at the die plate. The die plate temperature is the same between 1KD and 4KD (Fig. S4). The SME when using the 4KD screw is significantly higher than the SME when using the 1KD screw for every sample. This is because the number of kneading discs adds more mechanical energy to the dough. The die pressure in 4KD was lower than 1KD. This indicates that the dough viscosity in 1KD may be higher, considering the fact that the flow rate was similar for both screws (4.5 kg/h) (Wittek et al., 2021b; Cornet et al., 2022) and the fact that the die plate temperature is the same between 1KD and 4KD (Fig. S4).

This viscosity difference can be considered as the reason of the wedge length difference. The wedge length of POPI-1 and PPI in 1KD (Fig. 3B) is shorter than POPI-1 and PPI in 4 KD (Fig. 3A). This could be attributed to the fact that products made with the 4KD screw suffered from more disruption of the structure of the aggregated protein, which explains why the dough viscosity in 4KD is lower than 1KD, which is suggested by the lower die pressure (Wittek et al., 2021b; Cornet et al., 2022). When temperature decreases, the resulting viscosity increase is more significant for a highly viscous concentrated plant protein dispersion than for plant protein dispersion with low viscosity (Emin et al., 2017). A lower viscosity when entering the cooling die could lead to smaller viscosity increases upon cooling in the die, and as a result a more parabolic flow pattern will develop, before the structure sets. The velocity difference between the surface and the center of the cooling die creates a V-shape wedge (Wittek et al., 2021b; Min et al., 1984). However, POPI-2 and Non-emulsion in 4KD extrusion did not show longer wedge length than 1KD. This could be the result of wall slip (Sánchez et al., 2001; Denn, 2001), as for the 4KD samples, especially for POPI-2 and Non-emulsion, a lot of oil leakage was observed, which can create an oil film at the wall which acts as a slip layer. This could result in a more flattened flow profile in the die (i.e. plug flow).

Fig. 3B shows that the wedge length depends on the way the oil is added to the product in 1KD extrusion. The length of the wedge increased when oil was emulsified with POPI-1 < POPI-2 < PPI. The addition of only oil (Non-emulsion) resulted in a product with the longest wedge length (Fig. 3B).

There were no significant differences between extrudates when using the 4KD screw, except when using PPI as an emulsifier, which led to a longer wedge length. (Fig. 3A). In 4KD extrusion with POPI-1, POPI-2, and Non-emulsion, oil leakage from the structure was more significant than for PPI, which may have resulted in more wall slip for the former, and hence a shorter wedge length compared to PPI (Sánchez et al., 2001; Denn, 2001).

As the oil leakage in 1KD screw was significantly less than that in 4KD, we assume that less oil leakage causes less wall slip in 1KD. The order we observed in the wedge length (POPI-1 < POPI-2 < PPI, < Non-emulsion) appears to be correlated with the interfacial properties of each emulsion. In oscillatory shear, *G′*- value decreased in the order of POPI-1 > POPI-2 > PPI (Table 2), and in dilatational rheology the *E*_*d*_*’*-values increased in the order POPI-2 > POPI-1 > PPI. Apparently, the stiffer the oil-water interface is, the shorter the wedge length becomes. When droplets bind to the protein matrix, a stiffer interface can lead to a stiffer dough and higher dough viscosity, and thereby to a reduced wedge length (Ikenaga and Sagis, 2024). These rheological parameters will be discussed in the next sections.

#### 3.1.3 Texture analysis for extrudates

Products made with different screws had a significantly different stiffness. The maximum rupture strength in the fiber direction (Ft) of all products is higher in the 1KD screw extrusion than the 4KD screw. Also the maximum rupture strengths in the direction perpendicular to the fibers (Fl) of 1KD for POPI-2 and Non-emulsion are higher than those of 4KD. This indicates that fewer kneading discs tend to create a stiffer extrudate. More kneading elements probably disrupt the structure of the aggregates formed in the screw section to a higher degree. This leads to a lower viscosity and die pressure as described in the previous section, and is affecting the structure formation in the die, leading to lower stiffness.

The values for Ft and Fl of 4KD showed that the stiffness of the extrudate decreases in the order POPI-2 > POPI-1 ≈ PPI > Non-emulsion extrudate (Fig. 5B and C). We recall that PPI had the longest wedge length of these samples.

For the 1KD extrudates the values for Ft showed that POPI-1 extrudate is stiffer than the PPI and the Non-emulsion extrudate. These findings indicate the increase in wedge length for the 1KD samples (Fig. 3B) indeed seems to be related to the effects the emulsifiers have on the stiffness of the dough (see Sec. 3.2, Fig. 8).

#### 3.1.4 Wedge length and structure of extrudate without oil

**Fig. 6:**
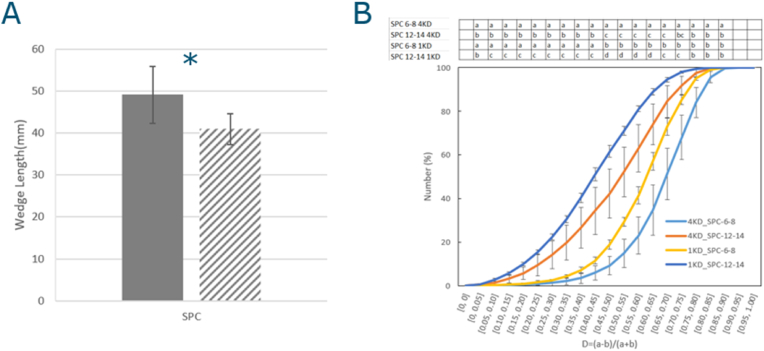
A: Wedge length of SPC control extrudate, solid bar: 4KD, shaded bar: 1KD B: Cummulative D-value distribution, calculated with Eq. (3), from image analysis comparing the 1KD with 4KD screw for SPC control extrudate, and comparing 6–8th FOV and 12–14th FOV. Statistical analysis: A; t.test ∗: P<0.05, ∗∗:P <0.01; B: One-way ANOVA, Tukey HSD test, P<0.05, values which were significantly different between FOVs and screw configuration are marked by different letters in the table above the figure.

We also observed the wedge length in extrudates using water (Table 4, Fig. 6A) prepared by both screws. The wedge length of the 4KD extrudate is significantly longer than that of the 1KD extrudate. The wedge length of the control sample is also longer than the wedge length of the extrudates with emulsion (Fig. 3A and B). Clearly, the addition of oil in general reduced the wedge length.

This difference in wedge length for the control between 1KD and 4KD, implied a difference in the protein structure between these samples. Multiphoton microscopy was used to quantify the structural differences between 1KD and 4KD. When we compare the 6–8th FOV and 12–14th FOV, the air pockets in the former are significantly more deformed than the air pockets in the 12–14th FOV, based on the D-values calculated with Eq. (3), for both 1KD and 4KD (Fig. 6B). The FOVs of the extrudate located near the wall of the cooling die, cooled down faster (Högg et al., 2017). This temperature difference affects the gradient in velocity across the cooling die, with the highest shear gradients located near the wall (Högg et al., 2017). This explains why air pockets near the surface deformed more. When we compare the effect of the screws, air pockets in the 4KD screw deformed more than those in the extrudates produced with the 1 KD screw, for both 6–8th and 12–14th FOVs. Chen et al., 2011 showed in a dead stop extrusion experiment that soy protein aggregates are formed by both 7S and 11S soy proteins in the screw section in high moisture extrusion processes, mostly by nono-covalent bonds, which are relatively easy to disrupt (Chen et al., 2011). The higher degree of disruption of these aggregates in the 4KD screw probably makes the dough less elastic and more viscous (i.e. more fluid-like) due to the breakage of protein aggregates (Chen et al., 2011) and disruption the extrudate structure, which we mentioned previously in the discussion of Fig. 4A in Sec. 3.1.3. This leads to a more parabolic flow profile in the cooling die, which leads to larger deformations of the air pockets, and as we mentioned before, also to longer wedge lengths.

#### 3.1.5 Structure difference between extrudates prepared with different emulsifiers

**Fig. 7:**
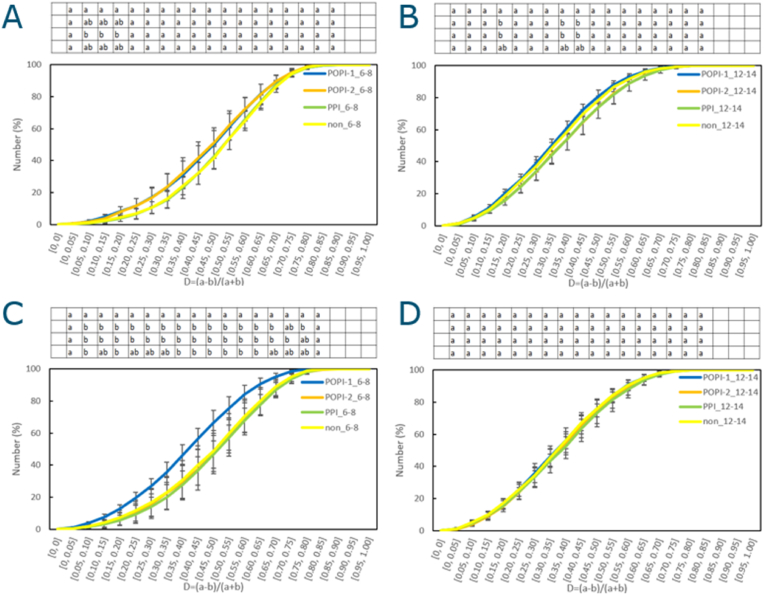
Cummulative D-value distribution at A: 6–8th FOV for 4KD B: 12–14th FOV for 4KD C: 6–8th FOV for1KD D: 12–14th FOV for 1KD (POPI-1: blue, POPI-2: orange, PPI: green, Non-emulsion: yellow) Statistical analysis result is shown in the table above the graph. (One-way ANOVA, Tukey HSD test, P<0.05, for each D-value) The order in the One-way ANOVA table above the graph is POPI-1, POPI-2, PPI, Non-emulsion from the top row.

Extrudates produced with emulsions with different emulsifiers were also imaged with multiphoton microscopy, to determine their structural differences. We again compared the D-values at the 6–8th FOV and 12–14th FOV (Fig. 7). The D-values for extrudates produced with the 4KD screw showed no significant difference between different emulsifiers (Fig. 7A and B). For the 1KD screw, the D-values at the 12–14th FOV in 1KD shows no significant difference between emulsifiers (Fig. 7D). However, the D-values at 6–8th FOV show a significant difference for POPI-1 (Fig. 7C), as its air pockets are clearly less deformed. This will be discussed further, together with additional rheological data in the following sections.

**Fig. 8:**
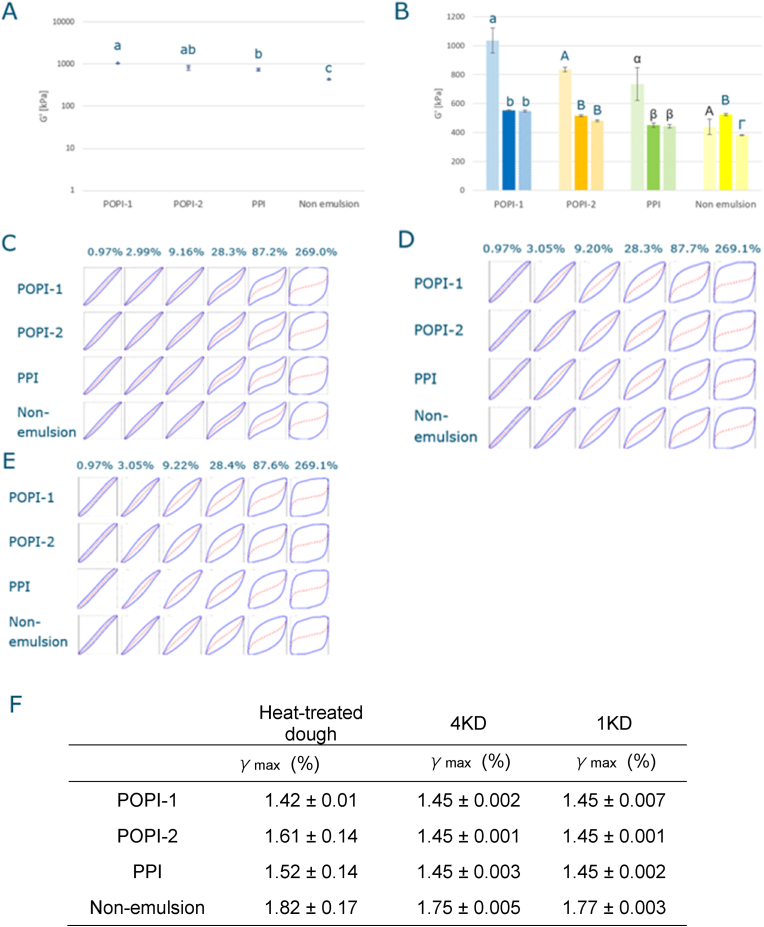
Shear rheological properties of heat-treated dough compared with extrudates prepared by the 1KD and 4KD screw. A: G′ for heat treated dough; Statistical analysis (One-way ANOVA, Tukey test, P<0.05) B: G’ (dotted bar: heat-treated dough, solid bar: 4KD extrudate, shaded bar: 1KD extrudate; colors: POPI-1: blue, POPI-2: orange, PPI: green, Non-emulsion: yellow); Statistical analysis (One-way ANOVA, Tukey test, P<0.05: letters used to indicate significant differences a, b: POPI-1, A, B: POPI-2, α, β: PPI, A,B,Γ: Non-emulsion) C: Normalized Lissajous plots for heat-treated dough; D: Normalized Lissajous plots for extrudate produced with the 1KD screw; E: Normalized Lissajous plots for extrudate produced with the 4KD screw; F: Maximum Linear Strain (γmax);.

### 3.2 Rheological measurements of dough

When comparing the wedge length for extrudates prepared with the 1KD screw (Fig. 3) and their maximum rupture strength (Fig. 5), we noticed that the wedge length increased, and the rupture strength decreased in the order POPI-1, POPI-2, and PPI. This order is similar to the order in which the interfacial shear storage modulus G_s_’ of the oil-water interface of the droplets decreases (Table 2). These observations suggest a correlation between the interfacial properties of the emulsion and dough properties (Ikenaga and Sagis, 2024). To establish if and how the stiffness of the oil-water interface affects the dough stiffness, we performed large amplitude oscillatory rheological measurements on doughs prepared using emulsions and compared it to a dough prepared by adding oil as a non-emulsion.

To separate the combined temperature and shear effects on dough during extrusion, we compared *G′* values of the extrudate (Fig. 5D) to *G′*-values of dough subjected to the same temperature profile of the extrusion process, thereby excluding the effects of shear alignment occurring in the die. The dough containing the POPI-1 emulsion showed the highest *G′*, and dough with the PPI stabilized emulsion showed the lowest *G′* among all dough samples. As the order of the surface stiffness of an oil droplet followed the same order (Table 2) (Ikenaga and Sagis, 2024), it seems that the emulsion's surface stiffness indeed affects the dough stiffness. The *G′* in the linear viscoelastic regime for the non-emulsion dough showed a significantly lower value than all of the doughs prepared with emulsions (Fig. 8A).

These results indicate that dough becomes stiffer when oil is added in the form of an emulsion. The dough is a protein matrix, and it is well-known that inclusions in such a matrix affects its rheological properties. Depending on the nature of the inclusions, they can lead to softening as well as stiffening of the matrix (Chen and Dickinson, 1999). This depends on the size of the inclusions, their volume fraction, their shape, their rigidity, and whether they are binding or non-binding to the matrix. Small, rigid and binding particles tend to increase the stiffness of the matrix, whereas large, soft, non-binding ones tend to weaken it. Here, the emulsions were prepared such that they have similar droplet size. Shape and volume fraction are also the same, and since the emulsifiers are all protein-based, their interactions with the matrix are likely also similar. The only difference between them is the interfacial stiffness they impart on the oil-water interface, and thereby the rigidity of the droplets. So, most likely through that effect POPI-1 gives a significantly stiffer dough than POPI-2, and PPI.

There is no significant difference between the maximum linear strain between the three emulsifiers, and also the normalized Lissajous plots show very little difference. The disruption of the dough structure appears to be mostly controlled by the protein-protein interactions.

Fig. 8B shows that the G′-values for the heat-treated dough samples were significantly higher than the values for all other samples. The maximum linear strain (%) did not show a difference between the heat-treated dough, and the extrudates prepared with the 1KD and 4KD screw for the three emulsifiers(Fig. 8F). When we compare the Lissajous plots, the heat treated-dough showed a mild strain hardening effect, around 28% strain (Fig. 8C), but this effect was not observed in the extrudates (Fig. 8D and E). These show a mild strain softening effect at around 3%, as evident from the slight levelling off of the loop towards maximum intracycle strain. The plot then continuously widens as strain increases, showing a gradual but progressive breakdown of the microstructure of the extrudates. These data indicate that without shearing during the heating cycle, a stiffer protein network is formed, implying that the extruder process itself has a big impact on the properties of the protein network formed. For the heated doughs, a much larger difference in *G′* can be observed when different emulsions were added, compared to the differences in both 1KD and 4KD extrudates. When oil is added in the form of an emulsion under the mild shear in heat-treated dough, avoiding coalescence (i.e. maintaining a small droplet size), plant proteins on the surface of the oil droplets are more likely to bind and to increase the stiffness of the matrix as aforementioned (Chen and Dickinson, 1999). However, the shear in the extruder increases the shear forces on the droplets, which can lead to elongation or even breakage of the interfacial layer, and subsequently more coalescence of the oil droplets. This leads to fewer but larger droplets, thereby decreasing the stiffening effect, and forming softer matrix. Even though the stiffness of the extrudate differs between extrudates produced with the 4KD and 1KD screws, both screw patterns added more mechanical energy to the dough and led to more coalescence of oil droplets than in the heat-treated dough where the shear on the dough is milder than in the extruder. In Non-emulsion addition, the stiffness of the structure was less affected by the shear than in samples with emulsion addition. For that mode of addition droplets tend to be larger from the start, and the extrudates are therefore softer (Chen and Dickinson, 1999). The additional shear imposed by the extruder therefore has a much smaller effect.

## 4 Conclusions

This study investigated the effects of extruder screw configuration (1 versus 4 kneading discs) and mode of oil-dosing (emulsion versus non-emulsion) on the amount of oil that can be retained in meat alternatives produced by high moisture extrusion, and on the textural properties of the extrudates. In emulsion-based dosing several plant-based protein extracts were tested as emulsifiers. It was found that in emulsion-based addition, both the protein source used as emulsifiers and the screw configuration have a large impact on oil incorporation and the stiffness of products.

The comparison of the 1KD and 4 KD (Fig. 4) configurations revealed that having fewer kneading discs led to less oil leakage, a shorter wedge length, and a higher rupture force. More kneading discs leads to more disruption of the structure of the protein matrix, leading to more coalescence of droplets, and a lower dough viscosity. A lower dough viscosity facilitates the development of a parabolic flow profile in the die, and hence results in a longer wedge length.

The interfacial properties of the emulsifiers also affected the dough and extrudate properties. In emulsion-based addition we observed a decrease of *G’* in the LVE in the order POPI-1, POPI-2, PPI. In that order the surface shear properties also decrease. PPI also has the weakest interface in dilatational rheology, so we see that mechanical properties have an effect on dough and extrudate properties, with weaker interfaces leading to softer products. Besides, stiffer interfaces also lead to more oil leakage and lower wedge length. A stiff interface is more brittle, and under the high shear conditions in an extruder this led to more coalescence. Considering the quality of meat alternatives, oil needs to be incorporated into the structure without causing leakage and with a positive effect on stiffness. Our work here shows that both screw configuration and the choice of emulsifier have a big impact on the properties of the final product. By optimizing these two aspects, it is possible to improve the quality of meat alternatives in the future.

## CRediT authorship contribution statement

**Shuzo Hashimoto:** Investigation, Formal analysis, Validation, Writing – original draft. **Naoya Ikenaga:** Investigation, Formal analysis, Validation, Writing – review & editing. **Atze Jan van der Goot:** Methodology, Validation, Writing – review & editing. **Leonard M.C. Sagis:** Conceptualisation, Methodology, Writing – review & editing, Supervision, Project administration.

## Declaration of competing interest

The authors declare the following financial interests/personal relationships which may be considered as potential competing interests:

Shuzo Hashimoto reports financial support was provided by Dutch 10.13039/501100004725Ministry of Economic Affairs. If there are other authors, they declare that they have no known competing financial interests or personal relationships that could have appeared to influence the work reported in this paper.

## Data Availability

Data will be made available on request.
