# Supplementary material for: Effects of screw configuration and interfacial properties on oil incorporation in high moisture extrusion
Source: Curr Res Food Sci. 2025 Jan 31;10:100989. doi: 10.1016/j.crfs.2025.100989 (PMC11847292; doi:10.1016/j.crfs.2025.100989)
Supplement: Multimedia component 1 [file mmc1.docx]

Supplemental figures


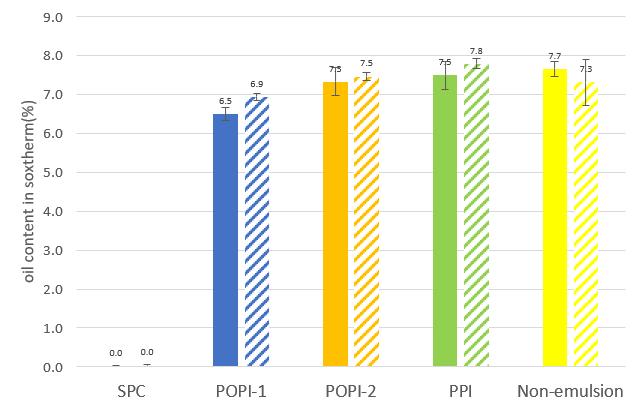


Fig S1. Oil content by means of Soxtherm extraction. (solid bar: 4KD, shaded bar: 1KD; SPC: gray, POPI-1: blue, POPI-2: orange, PPI: green, Non-emulsion: yellow)


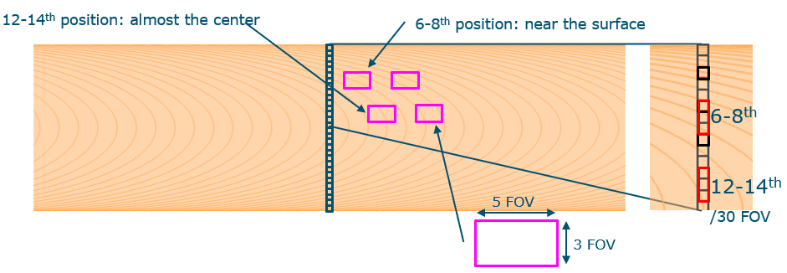

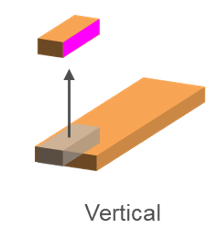


A

B


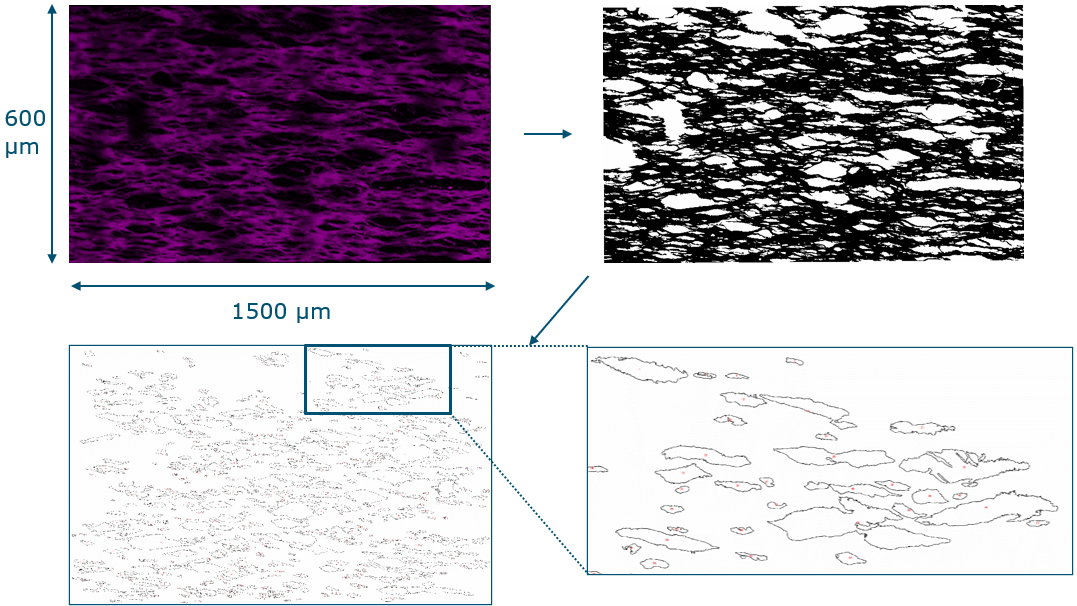


C

Fig S2. Multiphoton excitation microscopy A. Cutting method ; B. FOV levels; C Illustration of the image analysis (Conversion to greyscale and extraction of air pockets).


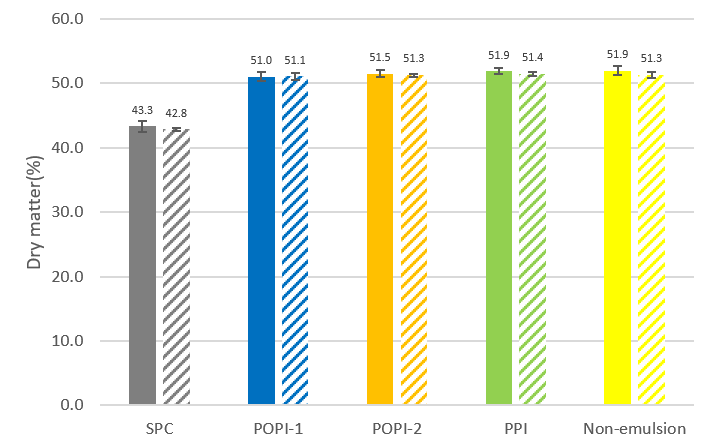


Fig S3. The dry matter content of extrudate (solid bar: 4KD, shaded bar: 1KD; SPC: gray, POPI-1: blue, POPI-2: orange, PPI: green, Non-emulsion: yellow)


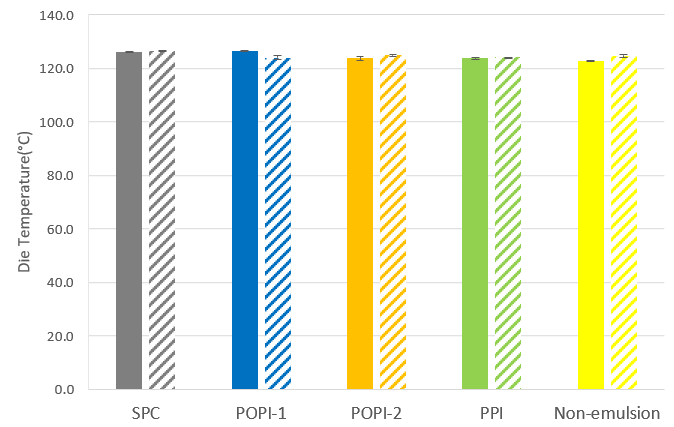


Fig S4. melting temperature (temperature of the dough at the die plate) (solid bar: 4KD, shaded bar: 1KD; SPC: gray, POPI-1: blue, POPI-2: orange, PPI: green, Non-emulsion: yellow)

|  |  |  |  | 4KD |  | 1KD |  |
| --- | --- | --- | --- | --- | --- | --- | --- |
|  |  |  |  | γmax (%) |  | γmax (%) |  |
|  | PoPI-1 |  |  | 1.55 ± 0.14 |  | 1.45 ± 0.01 |  |
|  | PoPI-2 |  |  | 1.55 ± 0.14 |  | 1.44 ± 0.00 |  |
|  | PPI |  |  | 1.55 ± 0.14 |  | 1.50 ± 0.11 |  |
|  | Non-emulsion |  |  | 1.76 ± 0.00 |  | 1.61 ± 0.16 |  |
|  |  |  |  |  |  |  |  |

Fig S5. Maximum strain(%) at the end of the linear regime in the Rheological measurements in the CCR.

Table S6. Composition of protein isolates. Protein content was measured by the Dumas method, fat content was measured with Soxhlet measurement using ether, and ash content was measured by the dry ash method. Carbohydrate content was calculated by subtracting protein, fat, and ash content from 100%. The measurement was performed in two replicates.

|  |  | Protein(wt %/DM) |  | Fat(wt%/DM) |  | Ash(wt%/DM) |  | Carbihydrate(wt%/DM) |
| --- | --- | --- | --- | --- | --- | --- | --- | --- |
| POPI-1 |  | 83.82 |  | 0.12 |  | 2.23 |  | 13.82 |
| POPI-2 |  | 86.67 |  | 0.12 |  | 0.06 |  | 13.15 |
| PPI |  | 75.65 |  | 0.41 |  | 4.91 |  | 19.03 |
